# Systemic *Spiroplasma turonicum* Infection Manifested as Knee Arthritis, France, 2025

**DOI:** 10.3201/eid3211.261001

**Published:** 2026-11

**Authors:** Matteo Vassallo, Sabrina Manni, Eleonore Delannoy, Johanna Azulay, Aurelie Canat, Laurene Lotte, Audrey Sindt, Anne-Flore Albertini, Pascal del Giudice

**Affiliations:** Unité de Recherche Clinique Cote d’Azur, Université Côte d’Azur, Nice, France (M. Vassallo); Cannes Hospital, Cannes, France (M. Vassallo, S. Manni, E. Delannoy, J. Azulay, A. Canat, L. Lotte, A. Sindt); Medipath, Mougins, France (A.-F. Albertini); Centre Hospitalier Intercommunal de Fréjus-Saint-Raphael, Frejus, France (P. del Guidice)

**Keywords:** Spiroplasma turonicum, bacteria, sepsis, arthritis, skin erythema, vector-borne infections, France

## Abstract

We report on an immunocompromised patient in southeast France who sought care for fever, knee arthritis, and skin erythema. Blood and joint samples were positive for *Spiroplasma turonicum*, an intracellular gram-negative bacterium generally found in plants, insects, and ticks. Clinicians should be aware of possible *S.* *turonicum* infection in immunocompromised patients.

*Spiroplasma* spp. are intracellular microorganisms belonging to the Mollicutes class and Spiroplasmataceae family, which also includes *Mycoplasma* spp. bacteria (*1*). *Spiroplasma* spp. bacteria require special substrates for growth in laboratory, and diagnosis often relies on use of genetic methods (*2*). Plants, insects, and ticks are known *Spiroplasma* bacteria reservoirs (*3*). 

Human infections are extremely rare, and *Spiroplasma* species differ according to vectors. For instance, 2 cases of *S. ixodetis* infections in Sweden were described in immunocompromised patients after tick exposure (*4*); 1 case of human infection with *S. apis*, a species generally associated with honeybees, was described in France after the patient was stung by a hornet-like insect (*5*); and 1 case was reported in Spain of human infection with *S. turonicum*, which generally colonizes in *Haematopota* spp. horseflies (*3*,*6*). We report a case of *S. turonicum* systemic infection associated with knee arthritis in an immunocompromised patient in France. The patient gave written consent to describe her case.

In November 2025, a 61-year-old woman sought care in Cannes Hospital, in southeast France, for a 10-day history of left knee pain. Her primary underlying condition was IgA lambda multiple myeloma, diagnosed in 2017, initially treated with autograft. Since 2024, she had been receiving teclistamab, a monoclonal antibody acting as a proteasome and CD38 inhibitor. 

At admission, the patient had an inflamed, painful left knee with functional impairment. Laboratory results showed elevated blood inflammatory markers, including C-reactive protein values at 83 mg/L (reference <5 mg/L), and serum protein electrophoresis revealed severe hypogammaglobulinemia (5.6 g/L, reference range 7.8–13.2 g/L), with undetectable IgA and IgM. She had inflammatory signs on the left knee, and positron emission tomography showed a hyperfixation in the popliteal region of the left leg. 

A knee aspiration revealed the presence of inflammatory cells (16,800 white cells/mL, most of which were polynuclear neutrophils). We cultured knee aspirate in blood culture bottles and incubated at 35°C in a BacT/ALERT 3D system (bioMérieux, https://www.biomerieux.com); after 48 hours, the cultures flagged as positive. Gram stain of cultures showed tiny gram-negative spiral-shaped bacteria. Subcultures on blood agar plates for 48 hours showed several small zones of β-hemolysis with no macroscopic colony growth. Matrix-assisted laser desorption/time-of-flight mass spectrometry using MALDI Biotyper (Bruker Daltonics GmbH, https://www.bruker.com) failed to identify the bacterium. At the same time, blood samples drawn from the patient were cultured and showed the same gram-negative spiral-shaped bacteria. 

Cytologic analysis of tissue samples collected during the arthroscopic lavage and debridement showed numerous polynuclear neutrophil cells, without any infiltration by cancer cells (Figure 1). PCR specific for *Campylobacter* spp. and *Helicobacter* spp. on the knee aspirate yielded negative results.

**Figure 1:**
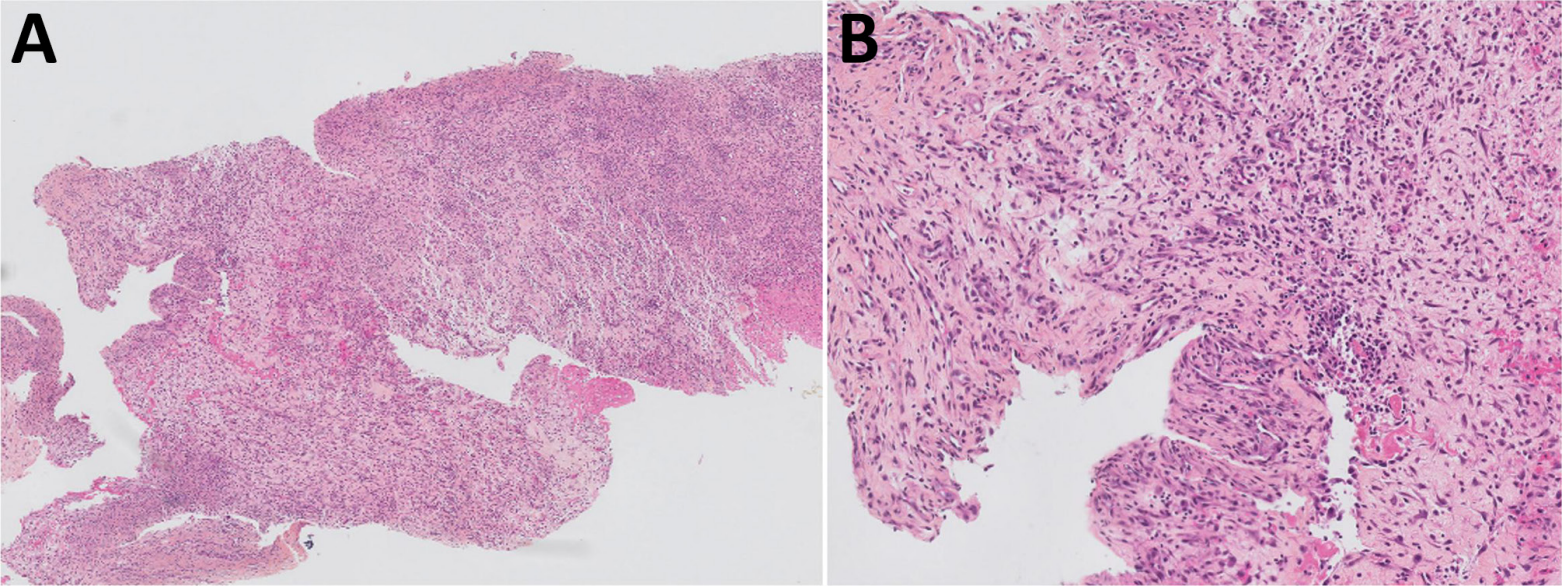
Histology of synovial sample of the left knee from an immunocompromised patient diagnosed with systemic *Spiroplasma turonicum* infection, France, 2025. Images show hematoxylin and eosin stain of polymorphous synovial inflammatory infiltrates. Polymorphous inflammatory granulomas are characterized by different white blood cell lineages in the knee synovial tissue. A) Original magnification ×2.5; B) original magnification ×5.

The patient was started on empirical carbapenems antibiotic therapy after an arthroscopic lavage and debridement of the popliteal nodular lesion. Blood inflammatory markers partially improved, but local inflammatory signs in the left knee persisted. A second arthroscopic lavage was performed 3 weeks after the first, and joint liquid analysis revealed the same inflammatory aspect; cultures were positive for gram-negative spiral-shaped bacteria but unrecognized by matrix-assisted laser desorption/time-of-flight mass spectrometry. After the surgery, the patient had several large asymptomatic annular skin erythema develop on her trunk, arms, and legs that lasted <48 hours (Figure 2).

**Figure 2:**
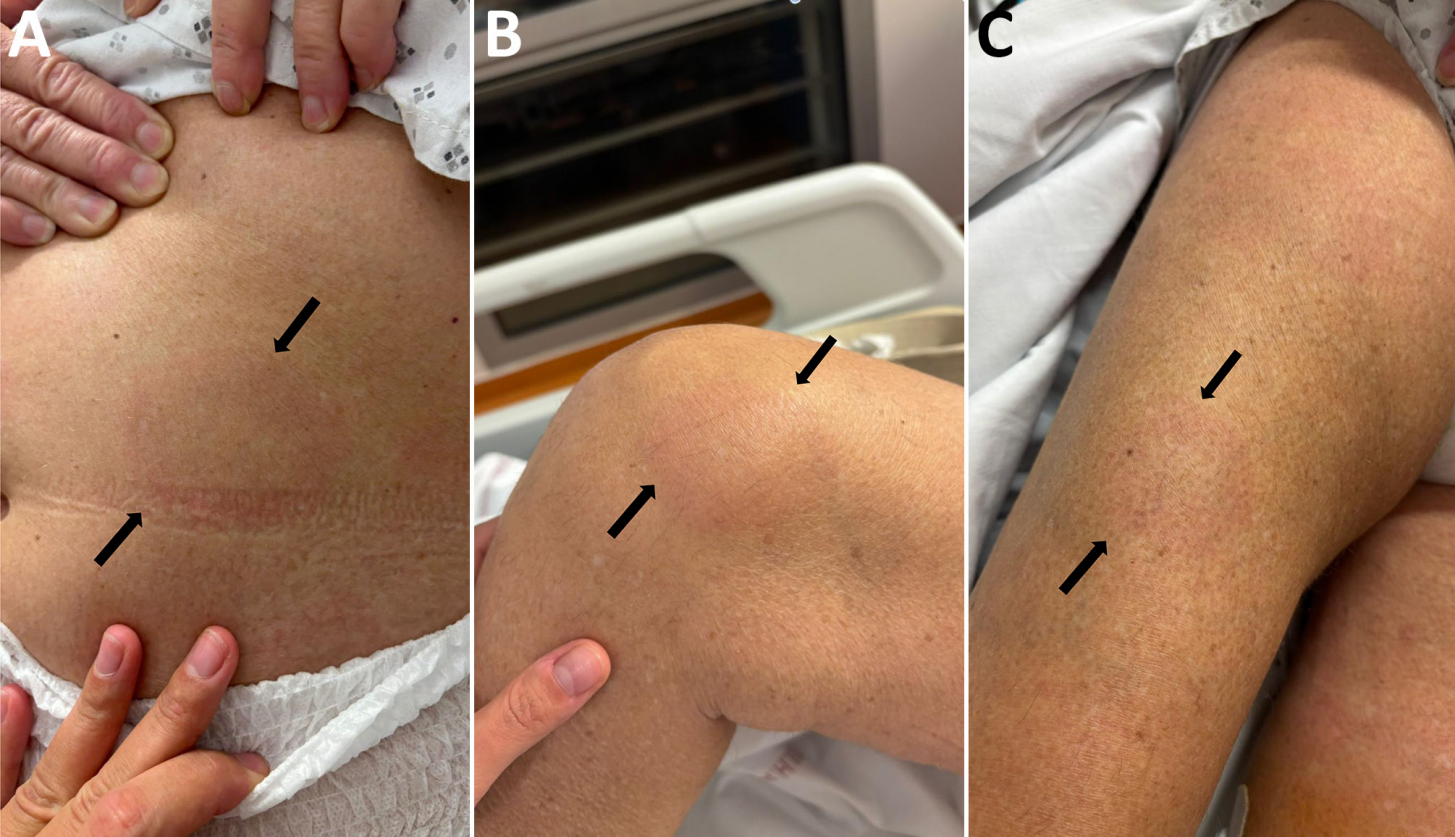
Skin annular erythema on an immunocompromised patient diagnosed with systemic *Spiroplasma turonicum* infection, France, 2025. After surgery, the patient had erythema (arrows) develop on the abdomen (A), knee (B), and thigh (C), which lasted <48 hours.

Finally, we performed 16S rRNA gene sequencing on the joint liquid and identified the bacterium as *S. turonicum.* According to previous literature (*4*,*6*), the patient was promptly started on a dual antibiotic therapy with doxycycline and levofloxacin, after which blood markers and local inflammatory signs progressively improved.

Because horseflies generally are the main *S. turonicum* bacteria vector, we asked the patient about the possible contact with horses. She cited a short stay in the inland region of Nice, in southeast France, with a visit to a horse ranch.

After 6 days of doxycycline and levofloxacin treatment and improved blood markers, the patient was discharged home and completed a 6-week course of antibiotics. She returned for outpatient follow-up 4 months after discharge; blood inflammatory markers were within reference ranges, and she had no signs of clinical relapse.

Skin manifestations, mainly as erythema multiforme, are known to be associated with *Mycoplasma pneumoniae* infections (*7*). Because *Spiroplasma* spp. and *Mycoplasma* spp. belong to the same family, we suggest that they could share the same pathophysiologic mechanisms associated with skin lesions. Moreover, arthritis due to *Spiroplasma* spp. has not previously been described, but *M. pneumoniae* has been isolated from joint fluid in some reported cases (*8*).

The patient in this case was severely immunocompromised because of multiple myeloma and the associated long-term treatment. Teclistamab targets the B-cell maturation antigen and causes a profound depletion of both malignant and normal long-lived plasma cells, resulting in prolonged hypogammaglobulinemia and a consequently substantial infectious burden (*9*,*10*).

In summary, we confirmed human *S. turonicum* infection in an immunocompromised patient with bacteremia, arthritis, and skin erythema. In severely immunosuppressed patients, clinicians and microbiologists should be aware of this very rare bacterial agent, which requires complex methods of identification and specific antibiotic treatment for resolution of infection.
